# The New York Quarantine

**Published:** 1888-01

**Authors:** 


THE NEW YORK QUARANTINE.—The State Board of Health of New York has forwarded to the Governor of the State a lengthy report on the New York Quarantine Station, which concludes as follows: "It is the unanimous opinion of those posted on such matters, that it would be difficult to imagine a worse state of affairs than now exists at the quarantine station. It is hard to realize in this age of civilization that the harbor of the city of New York should be so inadequately provided with facilities for the prevention and extinction of an epidemic. The State Board of Health does not consider it within its province to pronounce upon the actions of the quarantine authorities.—*Medical News.*

